# Influence of Sterilization and Preservation Procedures on the Integrity of Serum Protein-Coated Magnetic Nanoparticles

**DOI:** 10.3390/nano7120453

**Published:** 2017-12-15

**Authors:** Silvio Dutz, Stephanie Wojahn, Christine Gräfe, Andreas Weidner, Joachim H. Clement

**Affiliations:** 1 Institute of Biomedical Engineering and Informatics (BMTI), Technische Universität Ilmenau, Gustav-Kirchhoff-Strasse 2, D-98693 Ilmenau, Germany; stephanie.wojahn@tu-ilmenau.de (S.W.); Andreas.Weidner@tu-ilmenau.de (A.W.); 2 Department of Nano Biophotonics, Leibniz Institute of Photonic Technology (IPHT), A.-Einstein-Strasse 9, D-07745 Jena, Germany; 3 Department Hematology and Oncology, Jena University Hospital, Am Klinikum 1, D-07747 Jena, Germany; Christine.Graefe@med.uni-jena.de (C.G.); Joachim.Clement@med.uni-jena.de (J.H.C.)

**Keywords:** magnetic nanoparticles, superparamagnetic iron oxide nanoparticles, protein corona, UV sterilization

## Abstract

Protein-coated magnetic nanoparticles are promising candidates for various medical applications. Prior to their application into a biological system, one has to guarantee that the particle dispersions are free from pathogens or any other microbiologic contamination. Furthermore, to find entrance into clinical routine, the nanoparticle dispersions have to be storable for several months. In this study, we tested several procedures for sterilization and preservation of nanoparticle containing liquids on their influence on the integrity of the protein coating on the surface of these particles. For this, samples were treated by freezing, autoclaving, lyophilization, and ultraviolet (UV) irradiation, and characterized by means of dynamic light scattering, determination of surface potential, and gel electrophoresis afterwards. We found that the UV sterilization followed by lyophilization under the addition of polyethylene glycol are the most promising procedures for the preparation of sterilized long-term durable protein-coated magnetic nanoparticles. Ongoing work is focused on the optimization of used protocols for UV sterilization and lyophilization for further improvement of the storage time.

## 1. Introduction

Magnetic nanoparticles (MNP) are interesting tools for a variety of applications for diagnosis and therapy in medicine [1,2]. Prominent examples are magnetic drug targeting [3,4], magnetic particle hyperthermia [5,6] or magnetic particle imaging [7,8]. For this purpose, aqueous MNP dispersions (ferrofluids) have to be administered to the body, which requires a reliable sterilization of the ferrofluids to exclude any risk for the patient from pathogens within the fluid. Typically, this sterilization is performed by exposure to ultraviolet (UV) radiation or autoclaving [9]. 

When injecting MNP into a biological system (e.g., the peripheral blood of a patient), a layer made of different proteins present in the biological system (serving as a protein source) is formed on the surface of the MNP—the so-called protein corona [10,11]. This protein layer of a few nanometers [12] can be divided into a peripheral soft corona and a hard corona on the interface to the particles [13,14]. In our previous investigations [15,16] we could demonstrate that the formation of the corona occurs within seconds and the corona composition is furthermore influenced by the temperature at which corona formation takes place, as well as the duration of particle-protein interaction. It should be recalled here that for most engineered nanoparticles, their dilution in biological fluids (such as peripheral blood or fetal bovine serum) leads to particle aggregation [17,18,19]. It is also known that different particle properties (e.g., size and surface charge) exhibit an influence on protein corona formation and the resulting corona composition [20,21,22]. Furthermore, the protein corona plays a crucial role for the biological fate of the particles within the body [22,23,24]. The protein corona affects the cellular uptake profoundly and has an impact on the cell response, for example, cytotoxicity [25,26,27,28]. In a previous investigation we found that the strength of particle-cell interactions, as well as the kinetic ratio of internalized to adherent MNP in/on the cells, can be controlled by adjusting the amount of proteins bound to the MNP. Therefore, MNPs were equipped with a protein corona before introducing them into a biological system [29]. Resulting from all these findings, we conclude that protein-coated nanoparticles are interesting tools to study the interactions of nanoparticles with biological systems in in vitro and in vivo experiments. 

Similar to the common MNP, the protein-coated MNP also have to be sterilized prior to their in vitro/in vivo application. For particles smaller than 200 nm sterilization can be obtained by means of filtration. Unfortunately, this method is not suitable for sterilization of larger particles (>220 nm), because the membrane cut off for sterile filtration is 220 nm. Furthermore, a method for preservation of protein-coated MNP is needed, which guarantees storage for several months without an alteration of the integrity of the protein coating. In previous experiments we found a complete decay of the protein coating after nine days for storing in the fridge (4 °C). Up to now, standard procedures for sterilization and preservation of proteins are UV sterilization and lyophilization, respectively. Nevertheless, there are some critical steps which should be considered like freeze-concentration during the initial processes of lyophilization or secondary structure losses by crosslinking of aromatic groups [30].

In the recent years it was demonstrated that magnetic multicore nanoparticles show very promising magnetic properties for several medical applications [31]. Since these particles show a size above 200 nm, a sterilization by means of filtration is not possible. Therefore, the aim of the herein presented study is to establish procedures which allow a reliable sterilization and preservation of larger protein-coated MNP without any damaging effect on the integrity of the corona proteins. For this, protein-coated MNP were prepared as described before [16,32], treated by different procedures for sterilization and preservation, and characterized afterwards regarding possible damaging effects on the integrity of the protein coating.

## 2. Results and Discussion

### 2.1. Nanoparticle Properties

MNP were prepared by alkaline precipitation. The resulting particles consist of maghemite and show a mean core diameter of about 10 nm, as confirmed by X-ray diffraction and electron microscopy. After coating with diethylaminoethyl (DEAE) dextran, the mean hydrodynamic diameter determined by means of dynamic light scattering is about 190 nm. Therefore, a slight agglomeration of the cores prior to their protein coating can be assumed. The mean hydrodynamic diameter increased up to 250 nm after serum incubation. This might be due to the additional protein shell on the surface of the particles. An agglomeration of the particles during serum incubation seems not very probable when looking at the hydrodynamic diameters, but cannot be excluded completely. A second indication for a successful protein coating is the surface charge of the particles, which turned from +60 mV to −30 mV for particles prior and after incubation, respectively. 

These protein-coated particles were treated by different procedures for preservation and sterilization. The following sections describe the results of the particle characterization concerning their hydrodynamic size, surface charge, and the integrity of the protein coating after treatment; see Table 1. 

### 2.2. Freezing

Protein-coated MNP were frozen under two commonly used freezing conditions, at −15 °C (samples assigned “F”) and −80 °C (samples assigned “DF”). Protein-coated MNP samples were stored for one day (1d), two days (2d), one week (1w), two weeks (2w), four weeks (4w) or six weeks (6w). After thawing, samples were characterized immediately.

#### 2.2.1. Freezing at −15 °C

**Figure 1:**
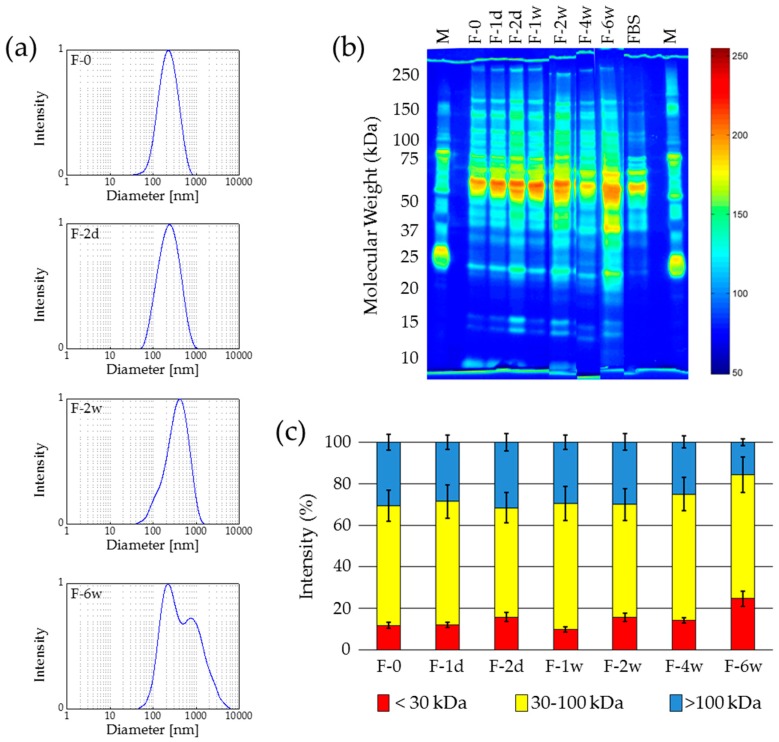
Freezing of protein-coated nanoparticles at −15 °C causes agglomeration during long-term storage. (**a**) DLS measurements exhibit a continuous increase of the hydrodynamic diameter with extended storage times; (**b**) Protein size distribution is shifted towards smaller sizes after several weeks of storage demonstrated by gel electrophoresis (silver staining and false color representation); (**c**) Variations of the protein size ranges over the storage period (based on (**b**)). *n* = 3, M—protein size marker.

The visual evaluation of the thawed samples showed that for storing times of two weeks and longer a significant change in the rheology of the fluid occurs. Larger agglomerates can be found in the samples and the viscosity decreases. These observations are confirmed by dynamic light scattering (DLS) measurements (Figure 1a). The original sample, F-0, as well as F-1d, shows a hydrodynamic diameter of about 260 nm. This value increased to 340 nm for F-2w and in F-6w agglomerates larger than 1000 nm can be detected. Furthermore, the polydispersity index (PDI) increased with the particle size simultaneously.

Despite the zeta potential provided a constant value of about −30 mV for all samples, it became obvious from the size measurements that, during the storage of the samples at a temperature of −15 °C, after two weeks a significant alteration of the integrity of the protein coating takes place. This assumption was confirmed by gel electrophoresis (SDS-PAGE).

Since the SDS-PAGE has to be carried out immediately after thawing of the samples, it was not possible to investigate all samples in parallel on one gel. Therefore, different runs of SDS-PAGE were performed during the study and the plots of the obtained lanes were merged (Figure 1b). The detailed investigation of the three groups of proteins contributing to the coating was defined: <30 kDa; 30–100 kDa; >100 kDa. The proportion of each group on the protein coating was calculated from an analysis of the grey value distribution in the raw greyscale image by means of ImageJ. The normalized optical grey value analysis by means of ImageJ shows a similar behaviour of the samples as observed by DLS (Figure 1c). Starting with sample F-2w, an increase of the amount of smaller proteins (<30 kDa) can be observed. For samples F-4w and F-6w a pronounced amount of the smaller proteins is present in the protein corona and the proportion of large proteins >100 kDa decreases. This behaviour can be interpreted as a degradation of the larger proteins to smaller fragments, which means a decay of the protein coating on the surface of the MNP. The degradation of the protein corona may then progressively liberate the metal core surface of the nanoparticles, and thus agglomeration is facilitated when these free metal sites come in contact [33].

In summary, freezing at −15 °C is not suitable for the long-term storage of protein-coated MNP. Short-term storage for up to one week might be possible.

#### 2.2.2. Deep-Freezing at −80 °C

**Figure 2:**
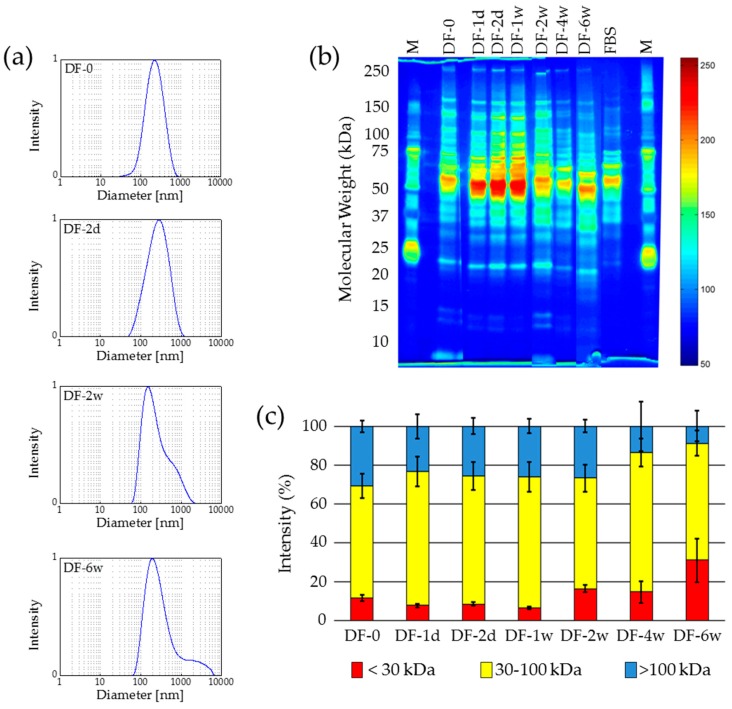
Deep-freezing of protein-coated nanoparticles at −80 °C affects the integrity of the protein corona during long-term storage. (**a**) DLS measurements confirm the occurrence of larger agglomerates with extended storage times; (**b**) Protein size distribution is shifted towards smaller sizes after several weeks of storage demonstrated by gel electrophoresis (silver staining and false color representation); (**c**) Variations of the protein size ranges over the storage period (based on (**b**)). *n* = 3, M—protein size marker.

Similar to the freezing experiments, deep-freezing caused an agglomeration of the MNP. Starting from a mean hydrodynamic diameter in DLS of 250 nm for the original sample (F-0), for all samples, but especially for longer storage times (two and six weeks) the occurrence of larger agglomerates was observed (Figure 2a). Furthermore, the zeta potential remained in the range from −29 mV to −30 mV for all samples indicating no alteration of the integrity of the protein coating.

For the investigation of the protein composition by means of SDS-PAGE, similar effects to those for freezing at −15 °C were observed. For storage times longer than two weeks, a significant alteration of the integrity of the protein coating was detected (Figure 2b and 2c).

In summary, the deep-freezing and storage of the samples at −80 °C revealed results similar to that at −15 °C. An agglomeration of protein-coated MNP was observed for storage at −80 °C similar to storage at −15 °C, and a remarkable alteration of the protein composition was proven for storage times of more than two weeks. Therefore, both the freezing and deep-freezing-based treatments presented here are no suitable procedures for the long-term storage of protein-coated MNP.

It remains to be seen whether a controlled automated freezing and thawing procedure as it is used for long-term storage of stem cell transplants may improve the outcome of long-term storage [34]. However, these procedures are very complicated and expensive.

### 2.3. Lyophilization

The protein-coated MNP samples were freeze-dried in the condition as prepared (samples assigned “Plain”) and after addition of polyethylene glycol (PEG; samples assigned “PEG”) or tetramethylammonium hydroxide (TMAH; samples assigned “TMAH”). PEG was used because it is suggested as cryoprotectant in combination with sugars [35]. The inherent sugars in serum were expected to be sufficient for this approach. After lyophilization, the dry powders were re-dispersed after one week (1w), three weeks (3w), or six weeks (6w) and characterized immediately. 

**Figure 3:**
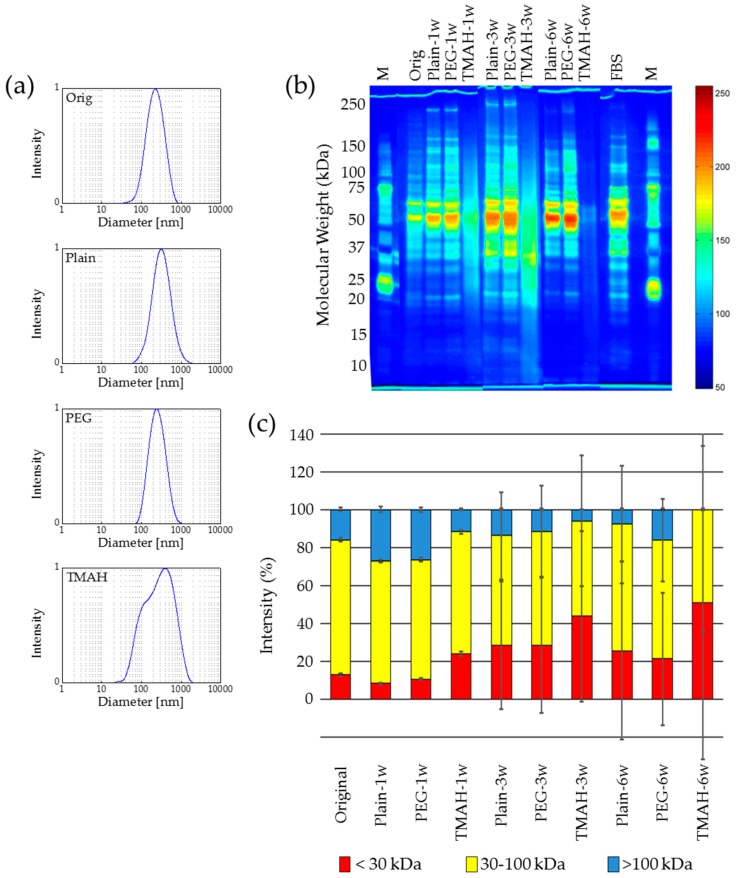
Lyophilization of protein-coated nanoparticles affects the integrity of the protein corona during long-term storage. (**a**) After storage of the dry powders for six weeks the DLS measurements show that the hydrodynamic diameter of re-suspended Plain and polyethylene glycol (PEG) samples is very similar to that of the original sample, but for tetramethylammonium hydroxide (TMAH) an agglomeration results; (**b**) Proteins are degraded during lyophilization, especially in the presence of TMAH, resulting in smaller sizes after several weeks of storage demonstrated by gel electrophoresis (silver staining and false color representation); (**c**) Variations of the protein size ranges over the storage period (based on (**b**)). *n* = 3, M—protein size marker.

For all three different re-dispersion time points a similar behavior in particle size and agglomeration was found in each single sample series (Plain, PEG, TMAH). The PEG samples show a similar size and size distribution after re-dispersion for all re-suspension time points like the original sample before drying. The Plain samples exhibit for all re-suspension time points a weak proportion of not re-dispersed agglomerates, and thus a slightly higher mean hydrodynamic diameter and PDI. When using TMAH as an additive, for all time points of re-suspension a large proportion of agglomerates remains after re-dispersion and the fluid is not stable against sedimentation, but also a certain fraction of very small fragments can be found in the sample (Figure 3a). This is due to the solubilizing activity of TMAH [36].

The analysis of the zeta potential revealed a constant value of about −30 mV for the original and all “Plain” and “PEG” samples of different storage times, whereas the zeta potential of all “TMAH” samples was in the range of −42 to −44 mV. From this zeta potential results an alteration of the protein integrity due to the addition of TMAH can be supposed.

Protein analysis showed that for a storage time of one week no remarkable changes in the integrity of the protein coating can be observed for “Plain” and “PEG” samples (Figure 3b and 3c). For TMAH-treated samples, already after one week an alteration of the protein integrity was observed. The amount of small proteins (<30 kDa) increased and the amount of larger proteins decreased, which is an indication for the degradation of the protein coating on the surface of the MNP. After a three-week storage period both, the sample Plain-3w and PEG-3w exhibit an increase in the low molecular weight fraction; nevertheless, the protein distribution remains unchanged.

After a storage time of six weeks, partial degradation of the larger proteins occurs and, in consequence, the fraction of small protein fragments and peptides increases. This might be due to stressors like freeze-concentration or pH-shift [37]. Furthermore, the large standard deviation in means of protein size distributions over repetitive experiments implies the pronounced variability in protein sizes upon re-dispersion of long-term stored samples.

In summary, the lyophilization of protein-coated MNP is a suitable method for the preservation of such particles for durations of up to one week. The re-dispersion of the dry powders can be improved by the addition of PEG prior the lyophilization. This phenomenon may be due to the increased stability of the protein-coated nanoparticles and the reduction of agglomeration [38]. For storage times longer than one week a partial degradation of the corona proteins can be observed.

### 2.4. Autoclaving

**Figure 4:**
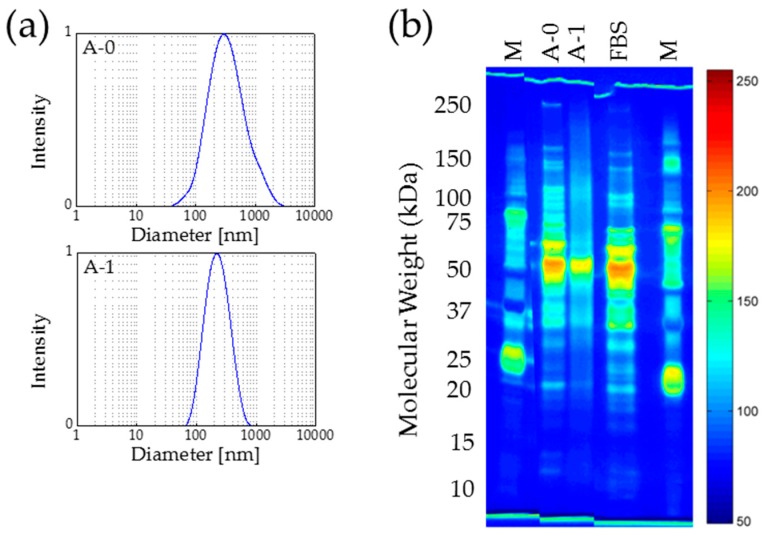
Autoclaving of protein-coated nanoparticles affects the protein corona dramatically. (**a**) DLS measurements show that the hydrodynamic diameter of the protein-coated nanoparticles decreases during treatment (**b**) Proteins are degraded during autoclaving demonstrated by gel electrophoresis (silver staining and false color representation). *n* = 3, M—protein size marker.

For the sterilization of protein-coated MNP by means of autoclaving, two groups of samples were prepared: samples without autoclaving (A-0) and autoclaved samples (A-1). The mean hydrodynamic diameter of 278 nm for A-0 decreased to 218 nm for the autoclaved samples A-1 (Figure 4a). The PDI decreased from 0.32 (A-0) to 0.24 (A-1) after the autoclaving process. On the one hand, this result might be interpreted as a decrease of the thickness of the protein corona due to coagulation of the proteins, which leads to a higher density of the proteins and thus a lower volume [39]. 

However, on the other hand, the observed changes in the analyzed parameter sets are in a similar range as those for the UV radiation exposure experiments. Therefore, the described changes in hydrodynamic particle sizes and particle size distributions are considered as not significant. A constant zeta potential of −31 mV for A-0, as well as A-1, was determined. Thus, the obtained results give no indication for a loss of protein content or alteration of protein integrity during autoclave sterilization of protein-coated MNP.

The interpretation that the components of the protein corona form a denser layer after autoclaving is supported by the SDS-PAGE results. After autoclaving the protein bands are no longer clearly separable but appear as a smear, especially in the high-molecular weight area (Figure 4b). The untreated sample (A-0) shows the typical pattern containing distinct protein bands for fetal bovine serum (FBS)-coated MNP, whereas the autoclaved sample (A-1) is mainly blurred and diffuse. All of the typical peaks are vanished, and only one agglomeration in the region of 55 kDa can be observed. Beside putative coagulation of the corona proteins, the denaturation and extensive degradation of the proteins may also contribute to these results.

In conclusion, the standard autoclaving protocol is not suitable for the sterilization of protein-coated MNP since the stability and integrity of the corona proteins is impaired.

### 2.5. UV Sterilization

**Figure 5:**
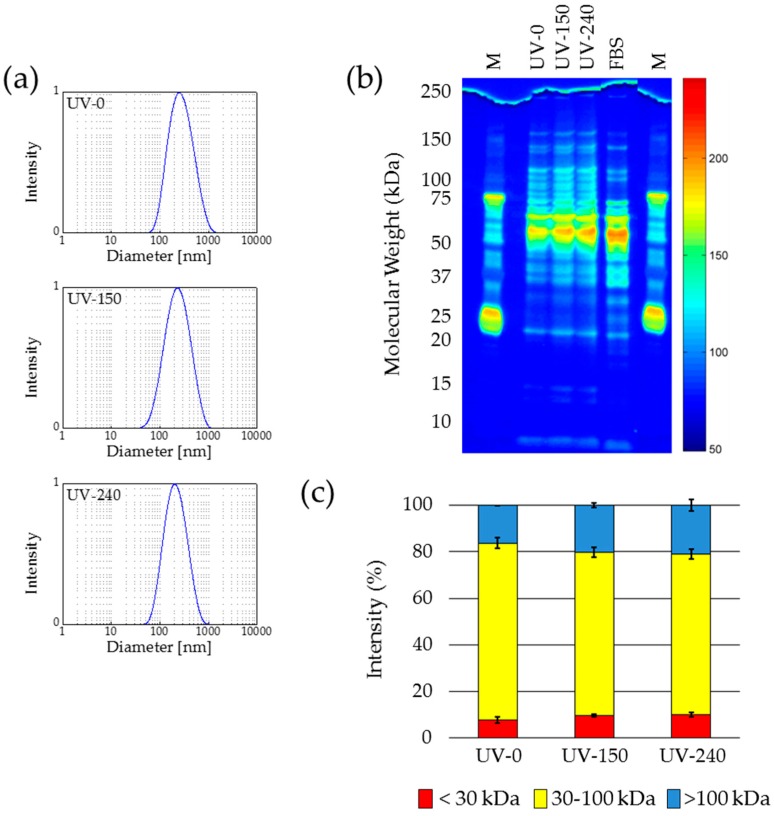
Ultraviolet (UV) sterilization of protein-coated nanoparticles does not affect the protein corona. (**a**) DLS measurements show that the hydrodynamic diameter of the protein-coated nanoparticles decreases with increasing treatment time (**b**) Protein size distribution is not altered as demonstrated by gel electrophoresis (silver staining and false color representation). (**c**) Variations of the protein size ranges over the storage period (based on (**b**)). *n* = 3, M—protein size marker.

The investigation of the samples without UV treatment (UV-0), samples with 150 min exposure time (UV-150), and samples with 240 min exposure time (UV-240) revealed no considerable changes in the mean hydrodynamic particle size, particle size distribution, as well as zeta potential due to the UV radiation (Figure 5a). The hydrodynamic particle size decreased from 257 nm for UV-0 to 218 nm for UV-150 and to 189 nm for UV-240. It seems that exposure of protein-coated MNP to UV radiation led to a reduction of the protein amount on particles surface with increasing exposure time. Since this hypothesis was not confirmed by the following characterization steps, the observed size decrease cannot be explained by protein degradation. Another reason for the decreasing particle size due to UV treatment might be a radiation-induced compaction of the proteins, as previously described by Durchschlag [39,40,41]. For the particle size distribution of all three samples a PDI in the range of 0.23 to 0.33 was determined. This indicates a relatively broad distribution of hydrodynamic diameters of the samples, which might be the reason for the observed variation in the mean particles sizes, too. For all three samples a zeta potential of about −30 mV was determined. All these measurements of physical properties indicate that there is no influence of UV exposure on protein amount on the surface of the MNP. This was confirmed by SDS-PAGE (Figure 5b,c).

Neither in the raw true-color image nor in the pseudo color greyscale image an evidence for any UV radiation-caused damage of the proteins could be observed. The pattern of the original sample (UV-0) and the patterns of the samples exposed to UV radiation (UV-150 and UV-240) looked very similar and showed their maximum intensities between 50 and 70 kDa, which is the typical range for albumin and its derivatives. The detailed investigation of the three groups of proteins contributing to the coating was defined: <30 kDa; 30–100 kDa; >100 kDa. The proportion of each group on the protein coating was calculated from an analysis of the grey value distribution in the raw greyscale image by means of ImageJ. In Figure 5c it is clear to see that in the frame of measurement accuracy, the samples before and after UV exposure revealed a comparable size distribution of the corona proteins. 

In conclusion, the exposure of a protein coating on MNP to UV radiation of 200–280 nm for up to 240 min causes no relevant change of protein content and integrity. Thus, the applied UV sterilization regime is suitable for the sterilization of protein-coated MNP.

## 3. Materials and Methods

### 3.1. Particle Preparation

The magnetic cores of the nanoparticles introduced in this paper were prepared similar to the well-known wet chemical precipitation methods [42,43], but using another alkaline medium and a slower reaction velocity. In detail, a 1 M NaHCO_3_ solution was slowly added under permanent stirring to a FeCl_2_/FeCl_3_ solution (total Fe-concentration: 0.625 M; Fe^2+^/Fe^3+^ ratio = 1/1.3). This procedure was stopped when the pH value reached 8. During this routine a brownish precipitate was formed. This precipitate was heated to 100 °C for 5 min and iron oxides with a spinel structure were formed under the release of CO_2_. To remove excess reaction products from the prepared particles they were washed with de-ionized water three times [44]. Nanoparticles were stored in de-ionized water at room temperature (RT).

For preparation of protein-coated MNP, the magnetic cores have to be incubated in a natural protein source, which leads to an accumulation of proteins on the surface of the MNP. For our studies, fetal bovine serum (FBS) was used as natural protein source. FBS was obtained from Biochrom GmbH (Berlin, Germany) (#S0115, Lot 1184C, tested for mycoplasma and viruses, tested for endotoxins) FBS was heat-inactivated at 56 °C for 1 h prior to use. FBS incubation of MNP was performed by water bath heating resulting in a homogeneous temperature distribution throughout the sample. For this, FBS was tempered at 37 °C in the water bath. A 15 mg pellet of prepared MNP from previously prepared suspensions was filled up with 2 mL of tempered FBS and kept at the same temperature for 10 min. During the time of incubation ultra-sonic treatment at the given temperature was carried out (S100H, Elmasonic, Singen, Germany) to re-disperse possible agglomerates. After 10 min the suspensions were taken out of the water bath and put on a magnet for magnetic separation, excess FBS was withdrawn and distilled water was added. The washed incubated nanoparticle suspensions were kept at 4 °C for short term storage prior to the following experiments [16].

### 3.2. Sterilization and Preservation

For a potential application of the protein-coated MNP in animals or human, one has to guarantee that the particles are free from biological pathogens or any biological contamination. After the sterilization it is important, to bring the particles into a condition which makes them storable for several weeks. For this purpose, freezing, lyophilization, autoclaving, and UV sterilization was applied to the protein-coated MNP. The following sections describe the applied procedures in more detail.

#### 3.2.1. Freezing

Freezing is a well-established procedure for the preservation of food but also of biological material. At low temperatures, the reproduction of bacteria is slowed and comes to a standstill at temperatures below 4 °C [45]. To check the influence of temperature on the integrity of the corona proteins after freezing preservation, the samples were frozen at −15 °C (freezing) and −80 °C (deep-freezing). Since the freezing time of the whole sample is a function of the temperature, freezing of the samples takes longer than deep-freezing. It is expected, that the freezing at −15 °C thus leads to the formation of larger ice crystals which might damage the integrity of the protein coating.

For this investigation, each time six samples of protein-coated MNP suspension of 1 mL were frozen at the two different temperatures and stored at the desired temperature for up to six weeks. After defined storage periods (1 day, 2 days, 1 week, 2 weeks, 4 weeks, 6 weeks) the frozen samples were thawed at RT and characterized immediately.

#### 3.2.2. Lyophilization

Lyophilization is also a well-established procedure for the long-term preservation of liquid protein-based pharmaceutical formulations [46]. During lyophilization, the sample is frozen at low temperature and, due to sublimation taking place at vacuum, water within the sample is removed. This leads to a complete cessation of all vital functions, as well as the reproduction of the pathogens since they need aqueous conditions to survive. After storage and before using the formulation, water is added again and after re-dispersion of the samples they should show the same properties like before. 

To investigate the influence of additives on the re-dispersion of the lyophilized samples, two different substances were added before lyophilization: tetramethylammonium hydroxide (TMAH) or polyethyleneglycol (PEG). After the lyophilization, the dry samples were stored at 4 °C and after certain storage periods (1 week, 3 weeks, 6 weeks) the samples were re-dispersed and the obtained suspensions characterized. For the re-dispersion, 1 mL of water was added and the samples were vortexed and treated with ultrasound.

#### 3.2.3. Autoclaving

For the sterilization of protein-coated MNP by means of autoclaving, a standard protocol was used [47]. For this, 1 mL of the protein-coated MNP suspension was placed into a Varioklav 25 T (Thermo Fisher Scientific, Waltham, MA, USA) and exposed to a temperature of 121 °C for 20 min at a pressure of 3 bar. This increased temperature leads to protein denaturation and damage of the DNA of potentially existing pathogens and causes their die-off. After a defined cooling down of the samples after the sterilization procedure, the samples were stored for some hours at 4 °C prior to their characterization.

#### 3.2.4. UV Sterilization

For the sterilization procedure, 1 mL of the protein-coated MNP fluid was exposed to a broadband UV-C radiation in the wavelength range from 200 to 280 nm. The optimum wavelength for the damage of nucleic acids, and thus the DNA of the pathogens, is 254 nm [47]. This DNA damage kills all potentially existing pathogens in the sample. 

The exposure time was adjusted for a first sample to be 150 min representing the minimum time for the sterilization, and for a second sample to be 240 min, representing a treatment time for sterilization including a safety margin. After the UV sterilization, the samples were stored for some hours at 4 °C prior to their characterization.

### 3.3. Particle Characterization

The magnetic properties were measured by vibrating sample magnetometry (VSM; Micromag 3900, Princeton Measurement Systems, Princeton, NJ, USA). Measurements were performed on liquid samples or dried powders. Concentration of MNP within the liquid samples and amount of proteins bound to particle surface were calculated from the obtained saturation magnetization. The overall magnetic behavior of the samples was derived from coercivity and relative remanence.

DLS was used to obtain information about degradation, agglomeration, or cross-linking of the protein-coated MNP due to the treatment for sterilization or preservation the MNP by investigating the size distribution of the particles. For these measurements, a Zetasizer (nanoZS, Malvern, UK) and appropriate software (Version 6.20) were used and particles’ size was derived from intensity-weighted scattering. Before the measurement, samples were diluted with a ratio of 1:30 in de-ionized water and treated in an ultrasonic bath. The medium viscosity and the relative dielectric constant were taken from water at 25 °C with 0.8872 cP and 78.5 cP, respectively. Measurements were performed in three consecutive runs and obtained values were averaged.

As shown before, the surface charge of protein-coated MNP might serve as an indicator for surface protein amount and composition [32]. Therefore, we used this parameter for a first check, whether the protein corona was influenced from the procedures to sterilize and preserve the corona coating. To determine the surface charge of the protein-coated MNP, the zeta potential is a valid and widely used parameter. For this, the Zetasizer was used again.

To evaluate, if the sterilization and preservation procedures damage the protein corona coating, the composition of the protein corona has to be determined before and after the treatment, respectively. The evaluation of the protein corona composition was carried out by means of SDS-PAGE, as described previously [32]. In brief, MNP samples were mixed with 4× XT Sample Buffer and 20× Reducing Agent (both from Bio-Rad, Munich, Germany) and heated up to 95 °C for 5 min to crack secondary and tertiary structure of proteins. Then, the denatured proteins were separated by molecular weight with polyacrylamide gel electrophoresis on a 4–12% Bis-Tris gel (Bio-Rad, Munich, Germany). After the run, the proteins were visualized by highly sensitive silver staining (SilverXpress Silver Staining Kit, Invitrogen, Heidelberg, Germany). Gel images were processed and analyzed by ImageJ (National Institutes of Health, Bethesda, MD, USA) [48]. As references, a molecular weight standard protein collection Kaleidoscope marker (Bio-Rad, Munich, Germany) and untreated FBS were used. Since the SDS-PAGE has to be carried out immediately after thawing or resuspension of the samples, it was not possible to investigate all samples in parallel on one gel. Therefore, different runs of SDS-PAGE were performed during the study and the plots of the obtained lanes were merged. The images of the original gels are provided in the Supplementary Materials, Figures S1–S6: images from the original silver-stained SDS-Polyacrylamide gels and corresponding false-color images.

## 4. Conclusions

With our investigations we could show that freezing at −15 °C allows storage of protein-coated MNP for up to one week. After that, a remarkable degradation of the larger proteins in combination with an agglomeration of the particles takes place. For deep-freezing at −80 °C agglomeration was observed, too, but also a degradation of the larger proteins after two weeks. The application of tightly controlled freezing procedures may achieve longer storability in the future. The addition of PEG to the samples prior to their lyophilization leads to well-dispersed fluids after re-suspension of the dry powders. Unfortunately, for storage times longer than one week a degradation of the proteins was observed. This storage period might be extended by using optimized storing conditions (e.g., lower temperature, oxygen exclusion, reduced humidity). Autoclaving by using a standard protocol embedded in a commercially available instrument is not suitable for the sterilization of protein-coated MNP since it damages the integrity of the protein coating dramatically. For the sterilization by means of UV radiation, we found no damaging effects for protein-coated MNP. 

In our experiments, we were very strict about the assessment of the alteration of the integrity of the protein coating. Even slight variations of the protein banding patterns elucidated by SDS-PAGE were interpreted as an alteration of the protein composition already, because they may be the consequence of disintegration or degradation and thus affect biological properties. This led us to calculate relatively short storage times without any visible change of the integrity of the protein coating. In prospective clinical applications such minor changes of the protein integrity may influence the interaction of the protein coating with the biological surrounding. Therefore, future studies are necessary to elucidate the treatment-dependent changes on the protein corona, as well as on the single protein level with regard to cytotoxicity and immunogenicity. Furthermore, the activity of enzymes as well as the availability of adaptor molecules and ligands has to be investigated in detail. Ongoing studies with cell-based and in vivo systems are focusing on this topic.

In conclusion, we state that the UV sterilization followed by lyophilization under addition of PEG are the most promising procedures for the preparation of sterilized long-term durable protein-coated MNP beyond the sterile filtration limit of 220 nm, see Table 2. The aim of the ongoing work is the optimization of the established protocols for UV sterilization and lyophilization in order to further improve the stability of the protein corona and the storage periods.

## Figures and Tables

**Table 1:** Summary of applied treatments and methods used for characterization. Dynamic light scattering (DLS); zeta potential (ZETA); sodium dodecyl sulphate polyacrylamide gel electrophoresis (SDS-PAGE).

Treatment Condition	Duration	Analysis		
		DLS	ZETA	SDS-PAGE
*Preservation*				
Freezing (−15 °C)	up to 6 weeks	X	X	X
Deep-Freezing (−80 °C)	up to 6 weeks	X	X	X
Lyophilization	up to 6 weeks	X	X	X
				
*Sterilization*				
Autoclaving	before/after	X	X	X
UV-Sterilization	before/after	X	X	X

**Table 2:** Summary of treatment methods and their suitability for sterilization and preservation of protein-coated magnetic nanoparticles.

Method	Suitable for Application	Remarks
UV-Sterilisation	yes	no major changes
Lyophilization	yes, with PEG as additive	degradation of large proteins with TMAH
Freezing (−15 °C)	only for short term storage	agglomeration of MNP, degradation of large proteins
Deep-freezing (−80 °C)	only for short term storage	agglomeration of MNP, degradation of large proteins
Autoclaving	no	degradation of proteins

## Supplementary Materials

The following are available online at http://www.mdpi.com/2079-4991/7/12/453/s1, Figures S1–S6: Original SDS-Polyacrylamide gels and false-color images.
