# Supplementary material for: Influence of Sterilization and Preservation Procedures on the Integrity of Serum Protein-Coated Magnetic Nanoparticles
Source: Nanomaterials (Basel). 2017 Dec 15;7(12):453. doi: 10.3390/nano7120453 (PMC5746942; doi:10.3390/nano7120453)
Supplement: Supplementary file 1 [file nanomaterials-07-00453-s001.pdf]

nanomaterials

MDPI

Supporting Information

Influence of Sterilization and Preservation Procedures on the Integrity of Serum Protein Coated Magnetic Nanoparticles

Silvio Dutz [1,2]*, Stephanie Wojahn [1] , Christine Gräfe [3], Andreas Weidner [1], and Joachim H. Clement [3]

[1] Institute of Biomedical Engineering and Informatics (BMTI), Technische Universität Ilmenau, Gustav-Kirchhoff-Strasse 2, D-98693 Ilmenau, Germany

[2] Department of Nano Biophotonics, Leibniz Institute of Photonic Technology (IPHT), A.-Einstein-Strasse 9, D-07745 Jena, Germany

[3] Department Hematology and Oncology, Jena University Hospital, Am Klinikum 1, D-07747 Jena, Germany

* Correspondence: silvio.dutz@tu-ilmenau.de; Tel.: +49 3677 691309

Academic Editor: Dr. Olivier Sandre

Received: date; Accepted: date; Published: date

Figures S-1 to S-6: Original SDS-Polyacrylamide gels and false-colour images

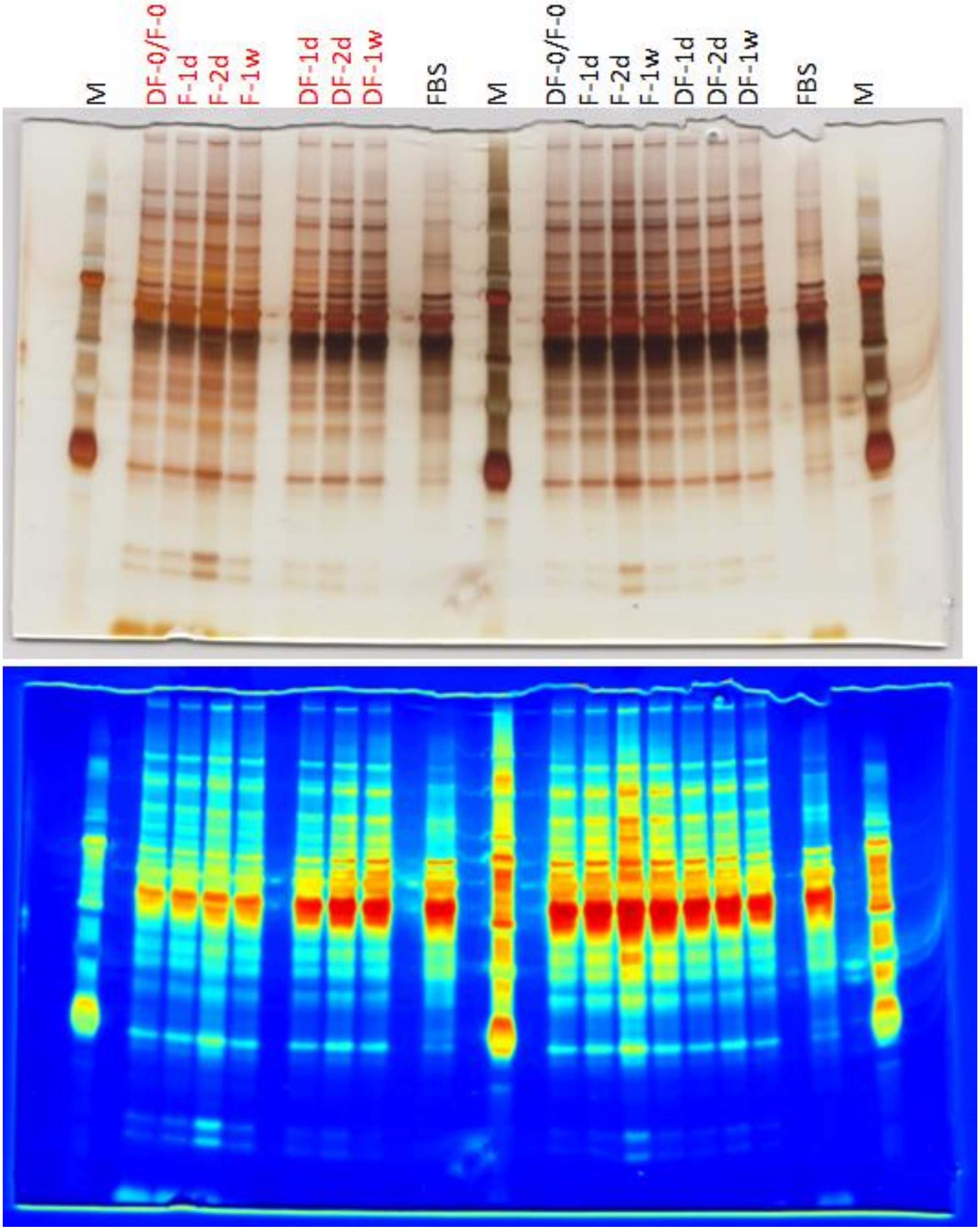

Figure S-1: Original silver-stained SDS-Polyacrylamide gel and false-colour image representing the protein patterns shown in Figure 1 (Freezing (F-) of protein-coated nanoparticles) and Figure 2 (Deep-freezing (DF-) of protein-coated nanoparticles). The relevant lines are highlighted in red.

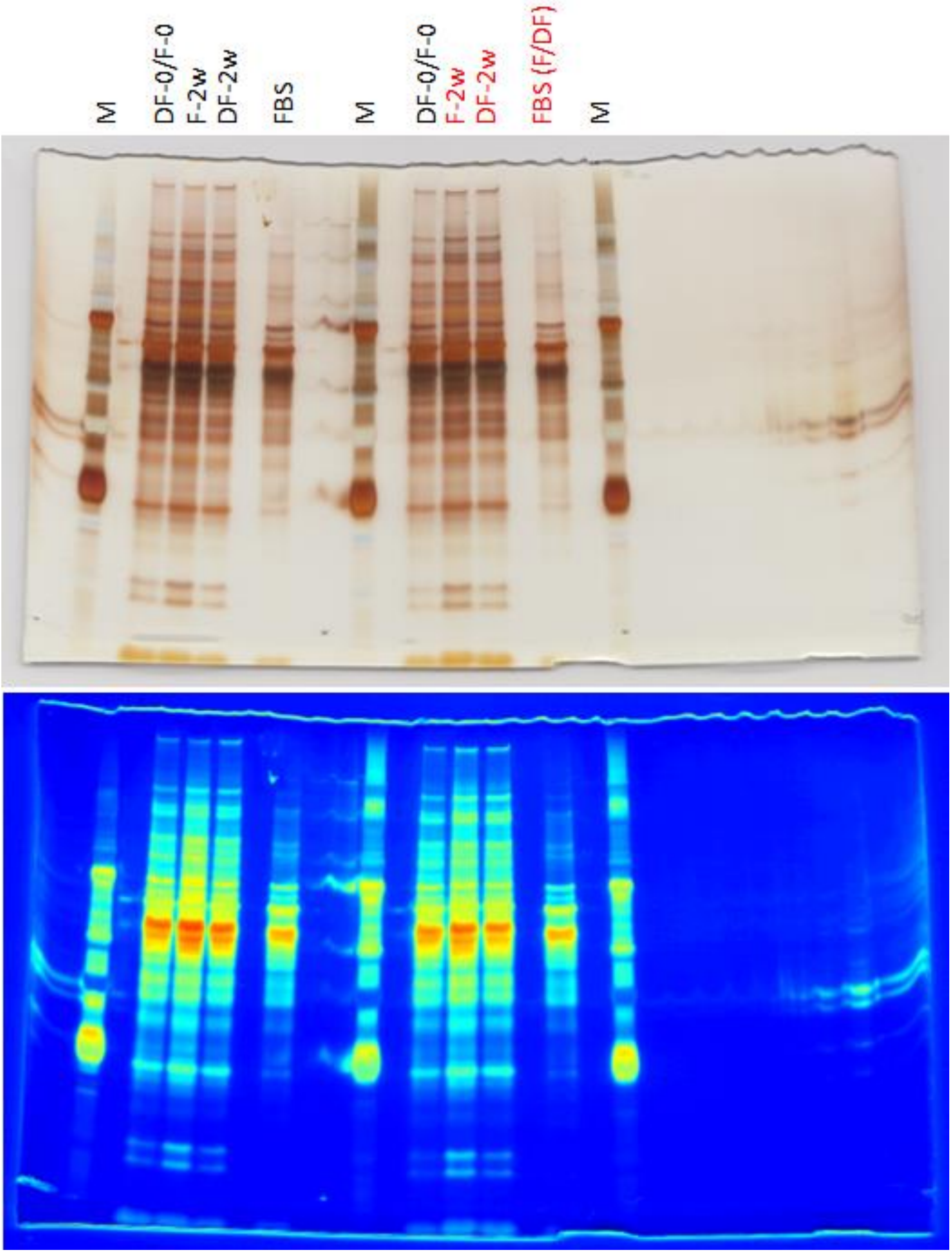

Figure S-2: Original silver-stained SDS-Polyacrylamide gel and false-colour image representing protein patterns shown in Figure 1 (Freezing (F-) of protein-coated nanoparticles) and Figure 2 (Deep-freezing (DF-) of protein-coated nanoparticles). The relevant lines are highlighted in red.

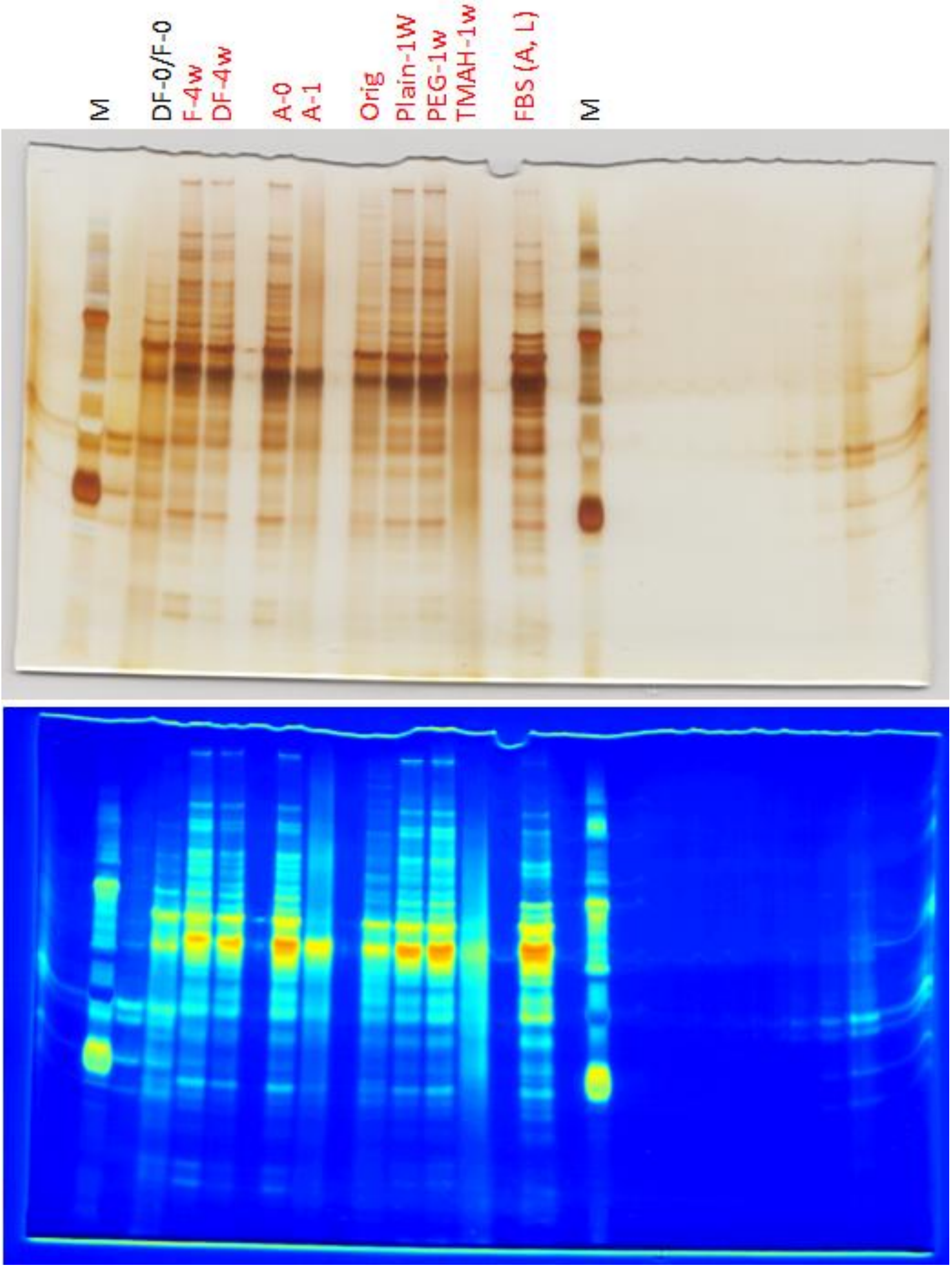

Figure S-3: Original silver-stained SDS-Polyacrylamide gel and false-colour image representing the protein patterns shown in Figure 1 (Freezing (F-) of protein-coated nanoparticles), Figure 2 (Deep-freezing (DF-) of protein-coated nanoparticles), Figure 3 (Lyophilization of protein-coated nanoparticles) and Figure 4 (Autoclaving of protein-coated nanoparticles). The relevant lines are highlighted in red.

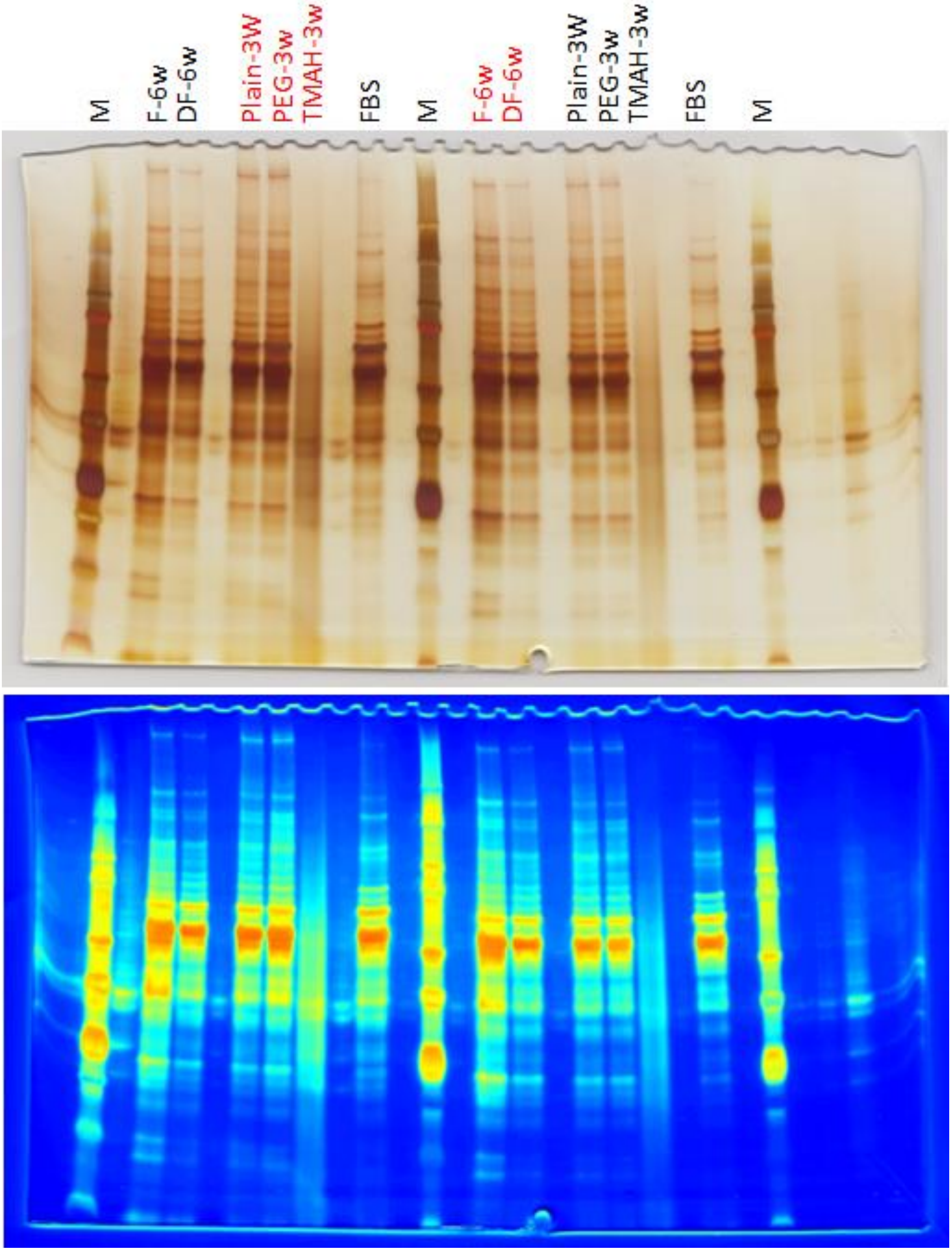

Figure S-4: Original silver-stained SDS-Polyacrylamide gel and false-colour image representing the protein patterns shown in Figure 1 (Freezing (F-) of protein-coated nanoparticles), Figure 2 (Deep-freezing (DF-) of protein-coated nanoparticles) and Figure 3 (Lyophilization of protein-coated nanoparticles). The relevant lines are highlighted in red.

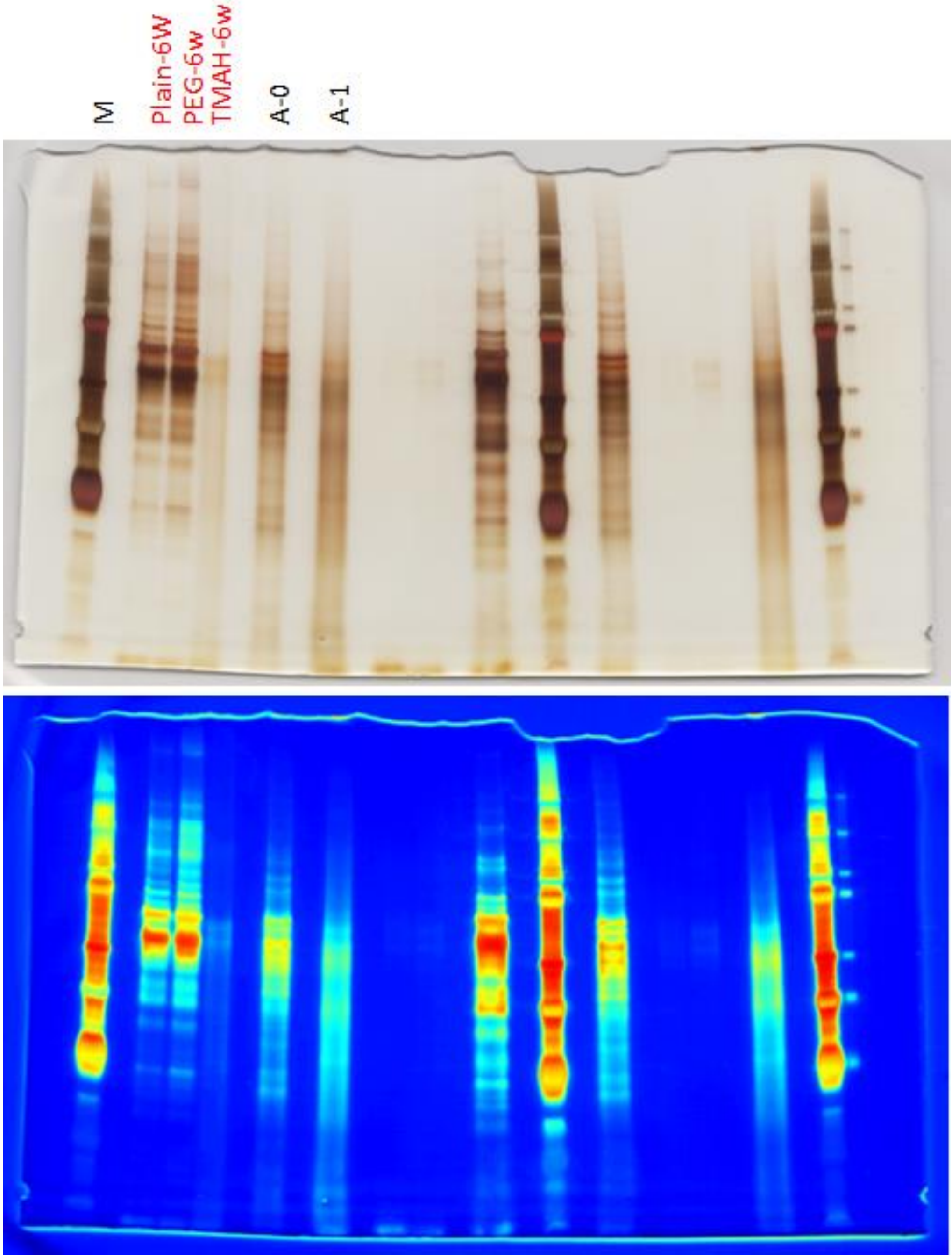

Figure S-5: Original silver-stained SDS-Polyacrylamide gel and false-colour image representing the protein patterns shown in Figure 3 (Lyophilization of protein-coated nanoparticles). The relevant lines are highlighted in red.

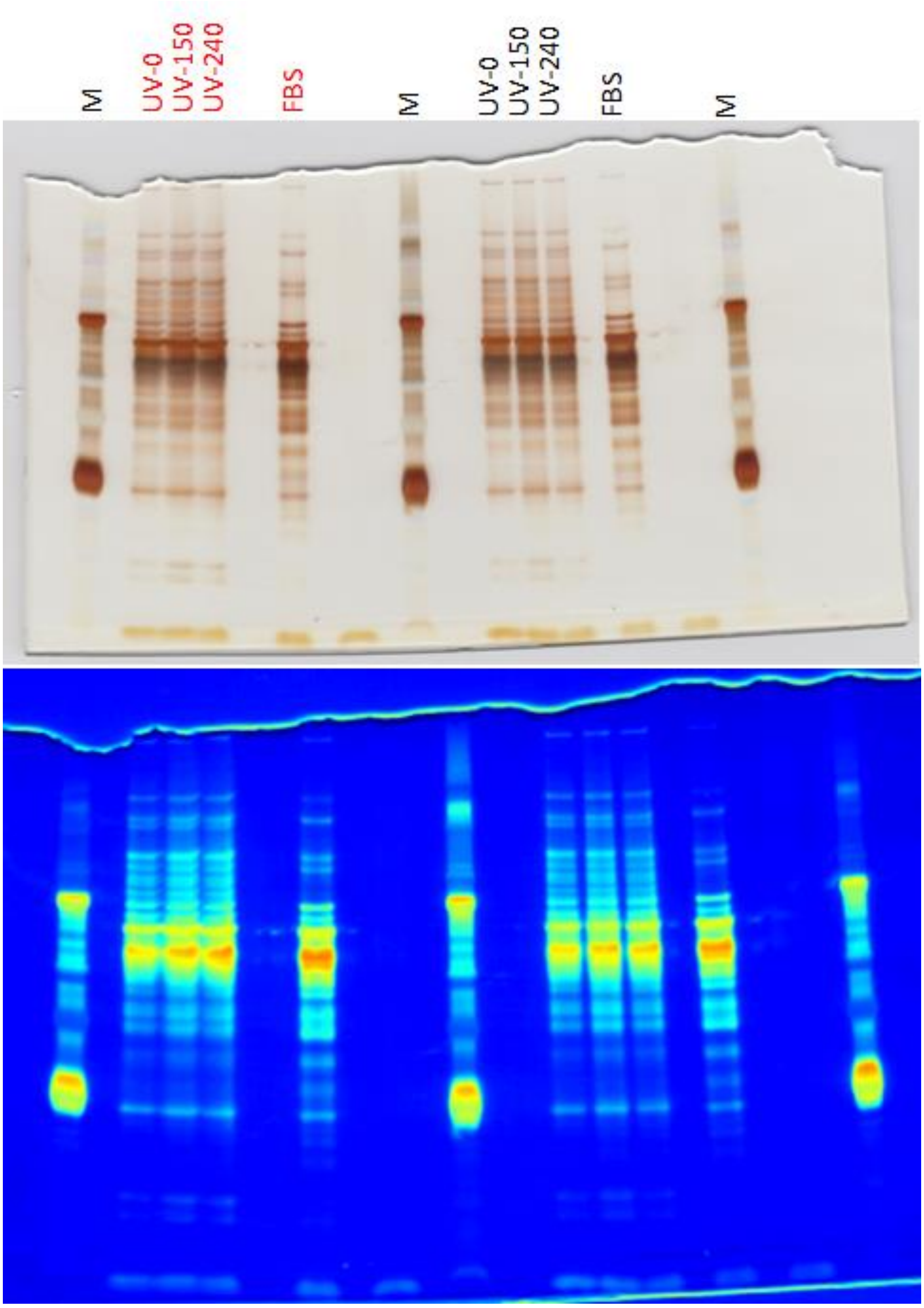

Figure S-6: Original silver-stained SDS-Polyacrylamide gel and false-colour image representing the protein patterns shown in Figure Figure 5 (UV-sterilization of protein-coated nanoparticles). The relevant lines are highlighted in red.
